# HistoMIL: A Python package for training multiple instance learning models on histopathology slides

**DOI:** 10.1016/j.isci.2023.108073

**Published:** 2023-09-27

**Authors:** Shi Pan, Maria Secrier

**Affiliations:** 1 Department of Genetics, Evolution and Environment, UCL Genetics Institute, University College London, London WC1E 6BT, UK

**Keywords:** Histology, Artificial intelligence, Machine learning

## Abstract

Hematoxylin and eosin (H&E) stained slides are widely used in disease diagnosis. Remarkable advances in deep learning have made it possible to detect complex molecular patterns in these histopathology slides, suggesting automated approaches could help inform pathologists’ decisions. Multiple instance learning (MIL) algorithms have shown promise in this context, outperforming transfer learning (TL) methods for various tasks, but their implementation and usage remains complex. We introduce HistoMIL, a Python package designed to streamline the implementation, training and inference process of MIL-based algorithms for computational pathologists and biomedical researchers. It integrates a self-supervised learning module for feature encoding, and a full pipeline encompassing TL and three MIL algorithms: ABMIL, DSMIL, and TransMIL. The PyTorch Lightning framework enables effortless customization and algorithm implementation. We illustrate HistoMIL’s capabilities by building predictive models for 2,487 cancer hallmark genes on breast cancer histology slides, achieving AUROC performances of up to 85%.

## Highlights

• HistoMIL: a package that streamlines deep learning tasks on histopathology slides
• Comprehensive preprocessing and efficient implementation of MIL algorithms
• Up to 85% AUROC in predicting hallmark gene expression in breast cancer H&E slides
• Cell cycle-related gene expression was most easily captured in pathology slides

## Graphical abstract

**Figure:**
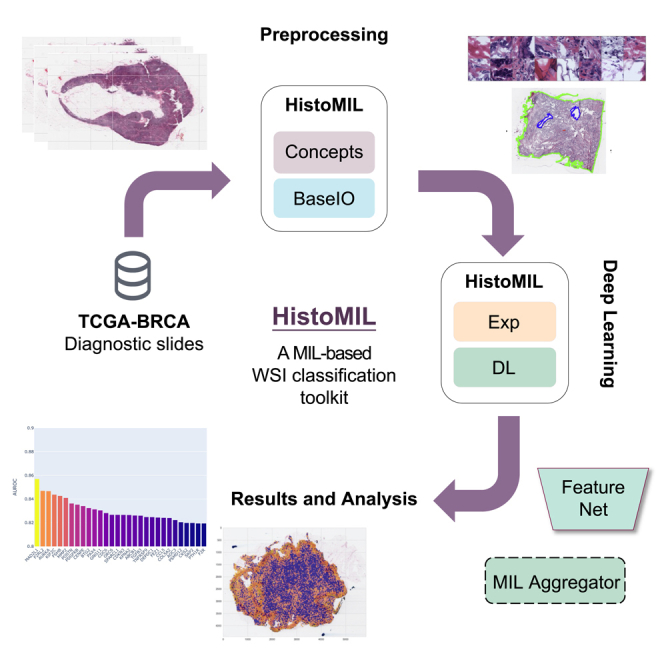

## Introduction

Histopathology slides stained with hematoxylin and eosin (H&E) are widely regarded as the gold standard for diagnosing cancer and other diseases. Deep learning (DL)^1^ based approaches have demonstrated remarkable potential for reproducing the workflows of human experts employing such slides in a variety of tasks, e.g., diagnosing cancer and classifying tumor types^2^^,^^3^; segmenting sub-regions at the pixel level to identify nuclei or tissue boundaries^4^^,^^5^; and predicting important clinical metrics such as survival^6^, recurrence rates^7^^,^^8^ and response to treatment.^9^ Several studies have shown that DL-based approaches can also predict more complex molecular characteristics from whole slide image (WSI) datasets, such as microsatellite instability, DNA damage repair deficiencies, mutations or gene expression patterns.^10^^,^^11^^,^^12^^,^^13^

Although transfer learning (TL) was initially widely used for WSI classification tasks, recent research has introduced multiple instance learning (MIL) as an alternative machine learning (ML) framework. MIL is designed to learn from bag-level labels rather than the more precise instance-level labels, and has been shown to outperform TL in certain tasks like survival prediction.^14^^,^^15^ However, implementing a MIL-based pipeline to predict molecular labels from WSI datasets presents significant challenges.

Digital pathology WSI datasets consist of large images scanned from original diagnostic tissue slides stained with H&E, often containing billions of pixels in a single file. This brings about specific challenges when applying MIL-based methods: (1) WSI files cannot be directly read by widely used image processing packages such as PIL,^16^ (2) classic architectures of a neural network are designed for lower resolution (i.e., 224 × 224 pixels^17^), and (3) loading an entire batch of WSIs during training is almost unmanageable and untraceable due to the limited GPU memory. There are various strategies to tackle these issues, and from a toolkit design perspective, they can be categorized into slide reading, preprocessing and ML oriented packages. While earlier implementations primarily focused on user-friendly WSI reading APIs, an increasing number of packages are now being designed to meet the requirements of ML algorithms. However, the existing pipelines do not fully exploit the capabilities of DL approaches.

***WSI reading packages/libraries*** such as OpenSlide,^18^ BioFormats,^19^ and HighDicom^20^ offer efficient tools for accessing raw WSI data formats, yet they exhibit shortcomings when integrated with neural network training. Notably, QuPath allows a basic ML pipeline with packages like scikit-learn^20^ through its graphical user interface, but it is not designed for DL or MIL algorithms. Pre-processing packages like PyHIST,^21^ deep-histopath,^22^ Multi_Scale_Tools,^23^ and ASAP^24^ include common processing steps like reading multi-scale information,^25^ tissue segmentation or patching. However, designing a comprehensive and broadly applicable ML-oriented package that encompasses basic components like WSI reading, patch extraction and color normalisation^26^^,^^27^ remains difficult. Some preprocessing packages, such as CLAM^28^ and deep-histopath,^22^ deliver quality preprocessing pipelines but may be restrictive when integrated with other algorithms or different datasets.

ML oriented packages like DeepMed,^27^ MONAI^29^ or TIAToolBox^25^ streamline the training process of DL models. For instance, MONAI extends PyTorch’s capabilities for medical data and imaging, providing specialized AI model architectures, transformations and utilities tailored for specific purposes. However, its core code does not specifically optimize for H&E data or various WSI formats. Packages like PathML^30^ aim to deliver richer content for their users by integrating more extensive models such as tissue segmentation. Also, researchers can easily and quickly train DL models on WSI datasets by using the TL protocol. For some of the open source packages, various useful tools have been integrated together, e.g., cell segmentation^30^ or graph aggregator.^25^ Packages like TIAToolBox also offer improved scalability through their module design and unit-testable code.^25^ However, a significant challenge in the context of TL arises when dealing with molecular-level labels lacking pixel-level annotations. This leads to reliance on pseudo-labels, potentially affecting model performance or generalization capabilities.^31^ For instance, consider a scenario where a tumor region is marked as exhibiting high expression of a particular gene that is unique to a subtype of T cells. In such cases, the model being used may end up learning intricate patterns for all cell populations in the tumor tissue, leading to the misclassification of all cells as positive cases. A robust feature encoder can also significantly influence the final results in target classification tasks. Self-Supervised Learning (SSL) has emerged as a prominent training paradigm that leverages the inherent data structure, negating the need for extra labeling. In the context of Whole Slide Imaging (WSI) classification, SSL holds the potential to create highly specialized feature encoders tailored for WSI datasets.^32^ Nevertheless, employing existing SSL packages requires multiple preprocessing steps for WSIs, PNG or JPEG conversion, followed by SSL training with invocation and processing. This essentially entails building a complete SSL pipeline alongside the core training process for WSI classification.

To address some of the challenges highlighted above, we introduce HistoMIL, a DL package based on PyTorch^33^^,^^34^ and PyTorch Lightning^35^ that simplifies the training of WSI-based classification models. HistoMIL provides a complete preprocessing pipeline, reducing the complexity of converting raw WSI data into a usable format for DL frameworks. We implement multiple MIL models that allow flexible training against different target labels, along with multiple SSL models that simplify the training of feature encoders. We demonstrate HistoMIL’s performance in predicting over 2,000 cancer-relevant molecular labels in breast cancer H&E slides from the Cancer Genome Atlas (TCGA). Additionally, we pinpoint specific pathways that can be effectively identified within histopathological tissue.

## Results

To facilitate the implementation of MIL-based workflows for classifying histopathology images based on specific molecular labels, we have developed a DL Python package entitled HistoMIL. HistoMIL leverages PyTorch and PyTorch Lightning to provide an efficient framework for all essential steps in DL tasks involving H&E slides. These steps encompass preprocessing slide data, training MIL and TL models on the processed dataset to predict molecular labels of interest, and visualizing the classifier performance and prediction results within the examined slides. Below, we showcase the features and capabilities of the package, accompanied by examples of its application to large-scale cancer datasets.

### Overview of the HistoMIL package

**Figure 1:**
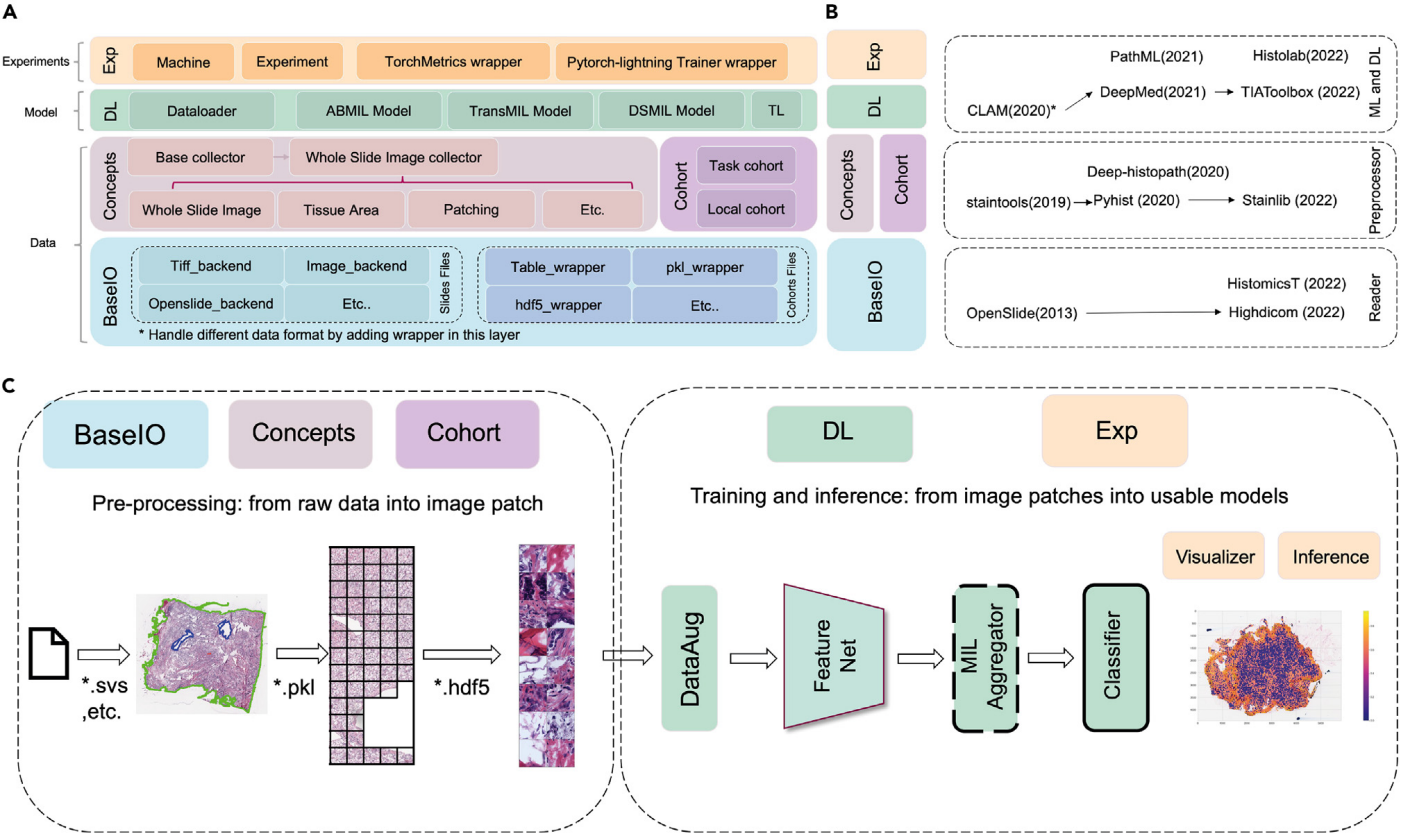
Overview of the HistoMIL package design and working pipeline (A) The diagram illustrates the relevant modules comprising HistoMIL and the logical structure organizing them. HistoMIL features four levels encompassing file reading, WSI-related processing modules, deep learning algorithms, and experiment management modules. (B) The relationship between HistoMIL modules and state-of-the-art libraries. The proposed package covers the entire workflow of WSI reading, preprocessing, and MIL algorithms, a design informed by the primary functionalities of other packages in the literature. (C) How HistoMIL’s various modules operate within an actual pipeline. We enumerate a typical processing workflow, including preprocessing, MIL training, and inference components. It is evident that different modules correspond to distinct functional parts within the process.

The HistoMIL library is structured into three tiers: **data**, **model**, and **experiment** (Figure 1A). The **data** level encompasses multiple data preprocessing steps, including WSI reading, tissue segmentation and patching. Similar to other ML-oriented packages, we incorporate functions for image normalization and feature extraction to store intermediate data and expedite model training. Additionally, we introduce a cohort level to handle metadata such as patient details, molecular labels, and other information. The design of HistoMIL aligns with the evolution of relevant packages in the existing literature (Figure 1B).

The **data** level draws inspiration from various WSI reading and processing packages. We introduce a universal reader class that allows users to customize different wrappers to access interfaces from other readers. This approach mitigates the limitation of relying solely on one reader package. For instance, OpenSlide,^18^ BioFormats,^19^ HighDicom^20^ are typical tools that provide a user-friendly API to handle WSI data formats. However, the Python interface of OpenSlide^18^ does not support certain formats, such as OME-TIFF.^18^ BioFormats^19^ and QuPath^20^ support a wider array of WSI formats but are heavily dependent on Java environments. In HistoMIL, users only need to adhere to our predefined abstract class, implementing a unified interface function for various reading libraries. This simplifies the conversion of diverse data types into a standard numpy matrix and associated metadata. Consequently, users have the flexibility to choose their preferred reading library based on individual requirements, thereby circumventing issues related to file formats. For the preprocessing steps, we have incorporated features inspired by the strengths of existing packages. For instance, we introduce a multi-threaded preprocessing design inspired by CLAM,^28^ and we allow users to pre-calculate features for the selected patches, a concept drawn from TIAToolBox,^25^ to expedite MIL model training.

The **model** level is divided into two key components: the backbone and the MIL method. Our backbone module seamlessly incorporates the interface of the timm PyTorch image model,^33^ enabling the downloading of various backbone network architectures and pre-trained parameters for feature extraction. This design philosophy is also present in other open source packages like DeepMed^27^ or TIAToolBox.^25^ However, these packages rely on TL methods, which consider only the original slide-level or patient-level labels, assigning them to each partitioned patch as training label information. In TL-based approaches, introducing pseudo-labels becomes necessary when pixel level labels are unavailable, but this introduces additional noise to the model training process. Training a batch of models may also pose challenges for packages like PathML,^30^ as models may learn misleading information from pseudo-labels and become trapped in local optima. In extreme cases, areas containing the cells of interest may be extremely small, with only a handful of patches containing genuinely relevant information. Our package implements multiple MIL methods (ABMIL,^31^ DSMIL,^32^ TransMIL^36^) alongside a baseline TL model. The entire algorithm implementation is based on PyTorch Lightning,^35^ enabling rapid training and fine-tuning of models for specific labels. Additionally, our package allows users to apply SSL protocols to train the feature extractor from scratch using only WSI datasets. Existing libraries often use pre-trained models from ImageNet as feature extractors. While recent research demonstrates that feature extractor networks trained with SSL on WSIs can enhance final performance,^14^,^32^ this feature is not supported by existing ML packages. Furthermore, HistoMIL includes pre-defined parameters as default settings, which allows users to quickly try out and scale-up an algorithm for different targets. Inspired by packages like stainlib,^26^ we have included some utility functions to handle data augmentation, a technique employed to ensure slides with different color ranges are transformed in a uniform way to enable meaningful comparisons.

At the **experiment** level, configuring different models for various datasets and hardware conditions becomes a straightforward task. For instance, researchers may wish to to gain a comprehensive understanding of how multiple biomarkers are spatially distributed by predicting these molecular labels from H&E slides. They might want to predict hundreds of target labels in an initial setting. Most existing packages lack the ability to efficiently scale up algorithms for multiple molecular labels, as they are not designed to deploy a batch of models simultaneously. In HistoMIL, researchers can effortlessly initialize a series of instances to explore the hyperparameter space defined by the customizable *para* class. A *Trainer* class instance will initialize a PyTorch Lightning module, adapting to hardware requirements automatically. Furthermore, third-party tuners like the Ray tuner^37^ can directly utilize these module instances, making it easy to discover the optimal model configuration. Figure 1C illustrates which modules are used at different stages of a process that employs a MIL algorithm.

**Figure 2:**
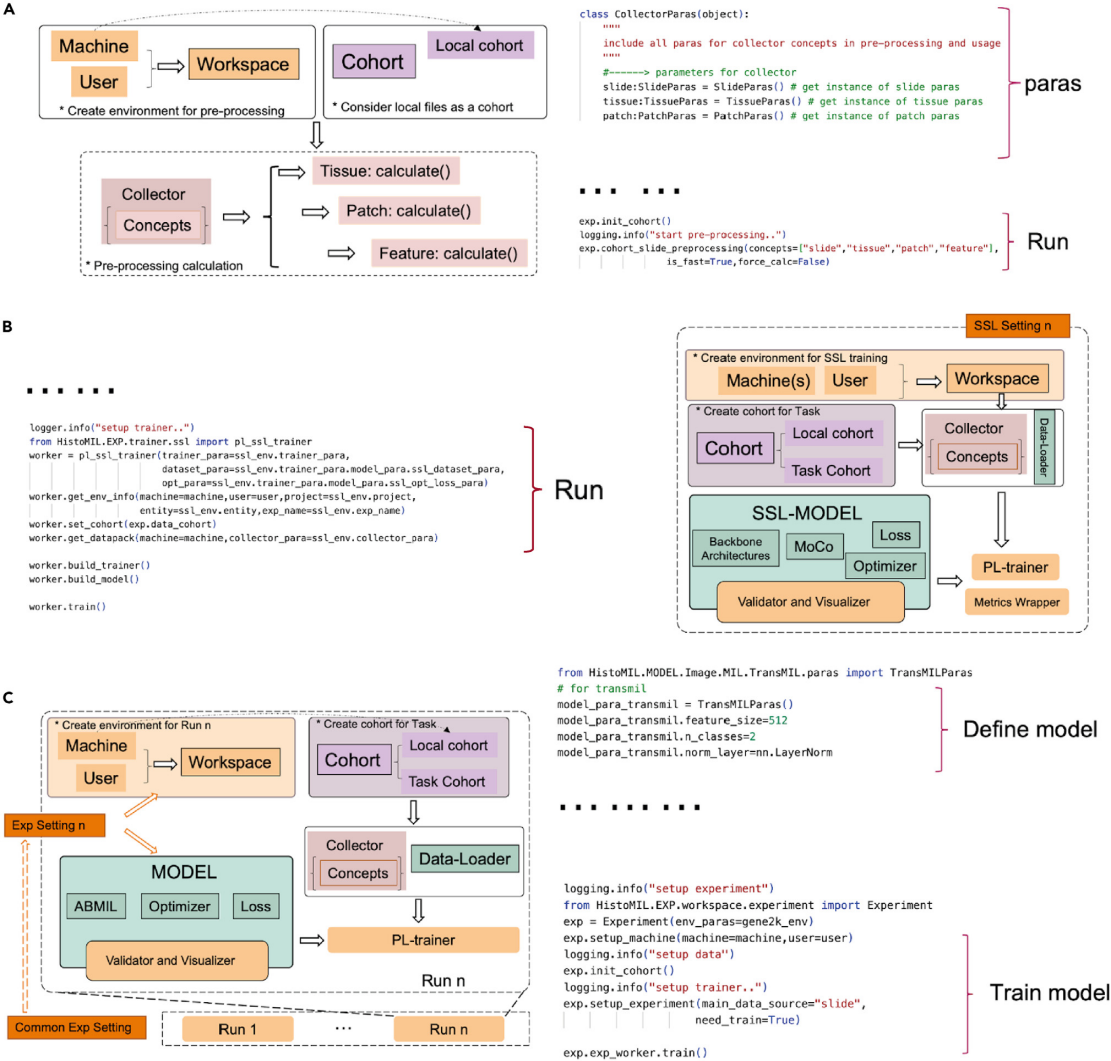
Diagram depicting three primary application scenarios for HistoMIL and their corresponding code blocks (A) The invocation method for HistoMIL during batch preprocessing. With predefined processing parameters, HistoMIL can process an entire dataset in one go using just three to four simple commands. (B) The code and calling logic for SSL training. HistoMIL facilitates SSL on WSI datasets by predefining SSL-related parameters and modifying the trainer accordingly. (C) The code and module calling process for MIL training. Compared to SSL, MIL adds adjustments to the model’s parameters while simplifying the training setup process. For users with access to a GPU server, numerous models can be trained simultaneously by configuring parameters in the bash file.

Details on the interplay between modules during preprocessing, SSL and MIL can be found in Figure 2. Moreover, Figure 2 demonstrates how the HistoMIL package streamlines complex pipelines with minimal code. Further information on the implementation of HistoMIL, including data-level design, model training and experimental setup, can be found in the STAR methods section.

### Applying HistoMIL to predict cancer hallmarks in H&E stained slides

In this section, we demonstrate the usability and scalability of HistoMIL in WSI-level prediction tasks employing state-of-the-art DL models. One clinically relevant task in cancer that has been recently made feasible by modern computational pathology is assessing the state of specific genes or entire molecular pathways directly within histopathology tissue slides using DL techniques.^10^^,^^38^ This rapid evaluation can aid in diagnosis, prognosis and treatment decisions. Typically, such assessments involve gene panel profiling or immunohistochemistry (IHC), but these tests introduce time delays and additional costs, as they are additional steps following visual inspection of routinely stained H&E sections. HistoMIL simplifies this process by providing a fast and efficient implementation for predicting diverse molecular labels in H&E-stained slides.

**Figure 3:**
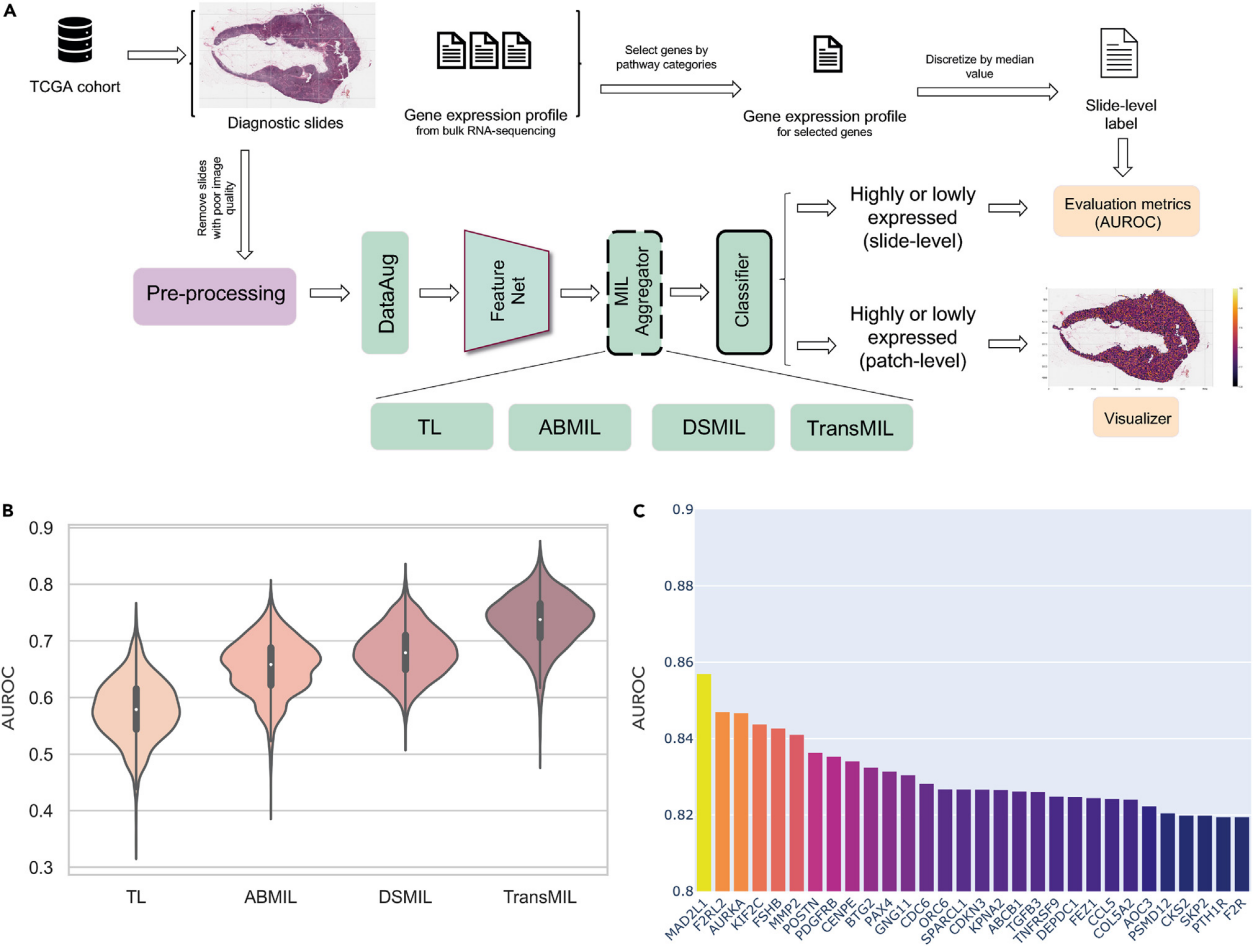
Experimental workflow and performance comparison (A) The diagram showcases the complete workflow for utilizing HistoMIL in predictive experiments. The raw data from TCGA-BRCA undergoes preprocessing, yielding image patches and associated labels suitable for MIL processing. The experimental task involves predicting gene expression, with the model’s performance displayed as AUROC. (B) Comparison of performance distribution among different algorithms in predicting the expression of 2,487 cancer-related genes. Each distribution contains 2,487 data points. The box centerlines depict the medians, and the edges depict the first/third quartiles. TransMIL exhibits superior performance relative to the other algorithms. (C) The top 30 genes with the highest AUROC scores. In the test set, the model’s accuracy in predicting gene expression levels (high or low) reaches up to ∼86%. See also Table S2.

Here, we showcase HistoMIL’s capacity to streamline the entire analysis workflow required to predict thousands of cancer hallmarks. We used 1,012 H&E-stained slides of breast cancer tissue and matched RNA-seq data from TCGA to train over 8,000 models for the classification of 2,487 cancer hallmark genes. First, we processed the WSI dataset using pre-defined preprocessing functions. This step involves extracting the tissue area from H&E histological images, generating patches automatically, and saving them as H5DF and image files. Subsequently, we trained the Feature Extractor Network using the SSL module and predicted the target gene expression labels using the built-in MIL algorithms. As depicted in Figure 3A, the implementation of the pipeline is simplified through the use of the generic interface provided by HistoMIL. This reduction in complexity significantly eases the workload for researchers aiming to expand these methods.

Despite the clear benefits of utilizing MIL algorithms for H&E-based predictions, researchers with limited coding experience may encounter difficulties when attempting to replicate MIL workflows, where slight variations in code may result in vastly different outcomes. Moreover, the complexity of handling high-dimensional histological data may discourage novice researchers from adopting this approach. In training our models, we adhered to the methodologies detailed in the original papers of TL,^27^ ABMIL,^31^ DSMIL^32^ and TransMIL,^36^ and employed the PyTorch Lightning wrapper designed by the HistoMIL library for the training process. This package simplifies these steps, making them accessible and ensuring reproducibility even for those lacking an in-depth understanding of MIL algorithms. For each algorithm, we trained over 2,000 models to predict the expression levels of selected cancer hallmark genes.

To demonstrate how HistoMIL handles the complex WSI processing steps, we have reproduced the pipeline in an instance notebook. This can be found in the *Notebooks* folder of the HistoMIL GitHub repository (see STAR methods). We have used code snippets and condensed the code to illustrate the usage of HistoMIL, underscoring its efficiency for WSI prediction tasks. Additionally, our model implementation includes options for computing an attention score or providing patch-level predictions, enabling MIL algorithms to predict the target labels for individual patches. In this particular example, we focused on WSI-level labels, where one expression value is available per gene and per slide. To expedite training and inference times, we did not perform additional data augmentation, yet the models were still able to successfully predict the target label.

Predicting gene expression labels in the benchmark TCGA breast cancer dataset showcases the potential of using HistoMIL on WSI datasets. All of the predictions are based only on WSI data. Our target labels encompass the expression levels of 2,487 different cancer hallmark genes derived from MSigDB, selected as a benchmark task to demonstrate the scalability of our package. We regarded this task as a typical weakly supervised learning challenge and opted for the TransMIL method to learn the aggregation function. We utilized the ResNet-18 pre-trained feature extractor as a backbone network, and all training procedures were executed on the PyTorch Lightning platform with early stopping.

Our experiments involved two training paradigms (TL and MIL) and a total of four different DL algorithms: TL, ABMIL,^31^ DSMIL,^32^ and TransMIL.^36^ The experimental outcomes consistently demonstrated that MIL algorithms generally outperformed TL algorithms when using the same feature encoder (Figure 3B). Furthermore, variations in performance were observed among different MIL algorithms, with a general trend of TransMIL > DSMIL > ABMIL. Considering the characteristics of these algorithms, it can be reasoned that the introduction of attention mechanisms and neighborhood information have played a significant role in driving these performance differences. Firstly, the slide-level attention mechanism could allow the algorithm to compare the importance of patches within a slide for classification. Additionally, TransMIL, which incorporates neighborhood information, has yielded the best results in our experiments. This is possibly due to the neighbor information capturing the broader changes in tissue structure, which may be relevant in the context of a certain gene being expressed or deactivated.

**Figure 4:**
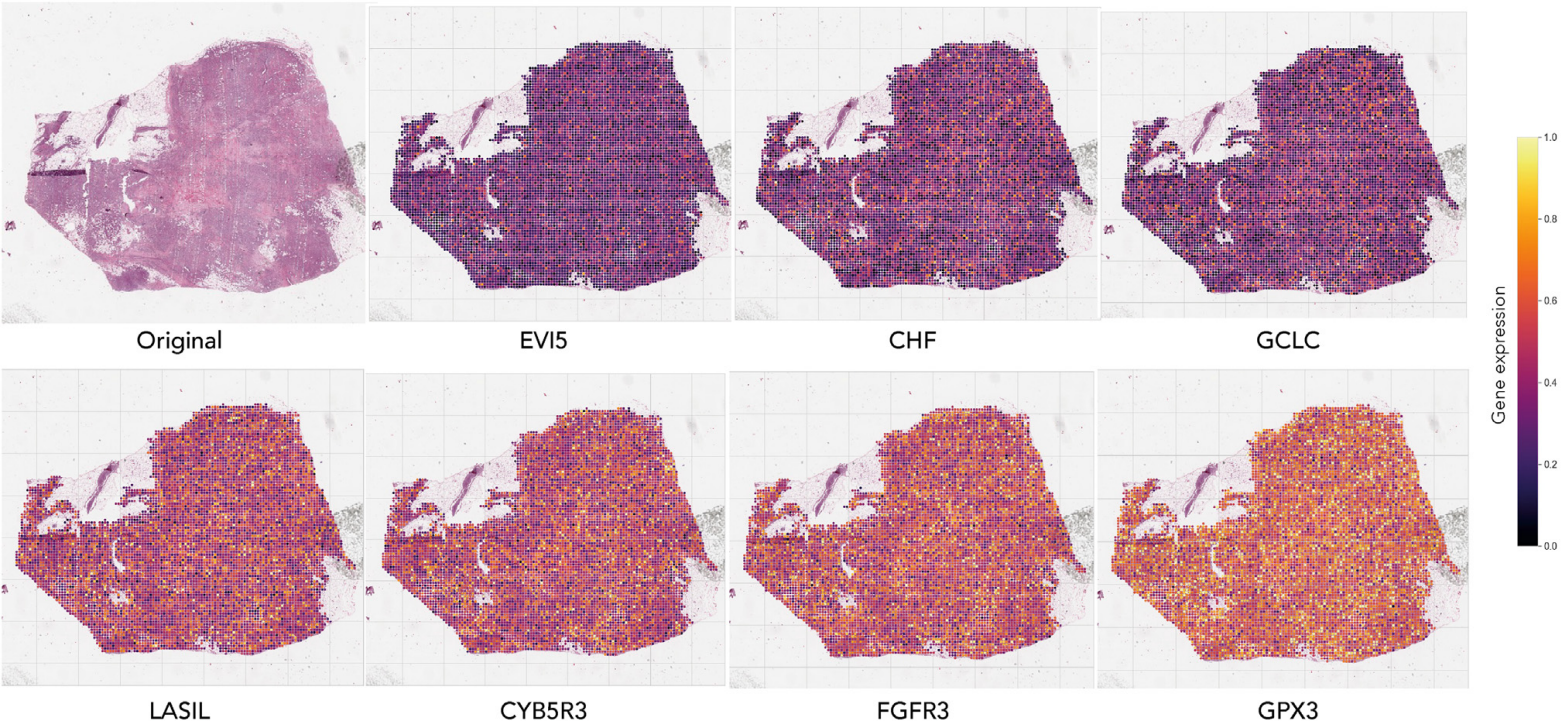
Model predictions for gene expression throughout the entire slide The first image on the left in the top row is the original tissue slide. The remaining heat maps in the first row demonstrate the spatial distribution of gene activity for selected lowly expressed genes that were predicted by the model. The heat maps in the second row exhibit the spatial distribution of gene activity for highly expressed genes that were accurately predicted by the model. The purple to yellow gradient indicates increasing levels of predicted gene expression per patch. Intratumor heterogeneity of gene expression can be observed, particularly for *LAS1L* or *CYB5R3*.

Among the top genes where different models demonstrated good performance metrics on the test set (Figure 3C, Table S2) were *MAD2L1, KIF2C* and *AURKA*. These are cell cycle regulators involved in spindle assembly and stabilization, and they promote chromosome segregation during mitosis.^39^^,^^40^^,^^41^ Other top-ranking genes such as *F2RL2* or *FSHB* are involved in G protein-coupled receptor signaling,^41^ while *MMP2* and *POSTN* are involved in matrix remodeling and cell adhesion.^42^^,^^43^ These highly relevant cancer-promoting processes would be expected to leave a clear morphological trace in the tissue. Therefore, the activity of these genes might be more easily identifiable due to linked changes in tumor cell morphology and tissue structure as seen in the H&E slides. In addition, since different MIL algorithms show similar performance in predicting the expression of these genes, it further indicates that the expression patterns of these genes in H&E slides have a certain predictability. These patterns can be easily captured throughout the entire slide using heatmap gradients of expression, thus informing on the spatial distribution of activity for a specific gene throughout the entire tissue (Figure 4).

Some genes, on the other hand, are more difficult to predict (Table S3). For instance, the performance of the TransMIL algorithm is poor when predicting the expression levels of *SPRR3*, a marker for terminal squamous cell differentiation linked with tumor progression in early stage breast cancers,^44^ and *PGAM2*, a gene involved in oxidative stress responses.^45^ This could be explained by the complexity of the regulatory processes driven by these genes which may not render clear morphological changes in the cells. This could also be compounded by tumor heterogeneity, e.g., if oxidative stress is present in only part of the cancer tissue. Furthermore, unlike *MAD2L1*, *F2RL2*, and *KIF2C*, genes such as *SPRR3* and *PGAM2* exhibit higher performance variability among different MIL algorithms, and their performance is normally lower than an AUROC of 65%.

**Figure 5:**
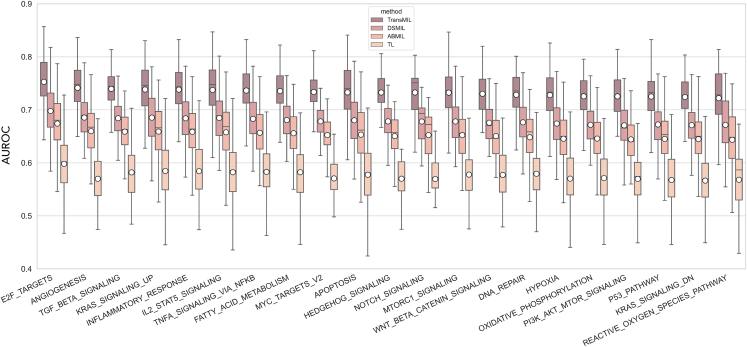
Performance of various algorithms on predicting pathway-level activity in cancer TransMIL, DSMIL, ABMIL and TL (Transfer Learning) algorithm performance is compared across selected pathways. The boxes depict the AUROC distributions for each algorithm, colored according to the legend. White circles represent the median values, while black lines indicate the mean. The edges of the boxes depict the first/third quartiles and the whiskers depict the minima/maxima. The different hallmark pathway groups are arranged from left to right in descending order of median values according to the TransMIL algorithm.

To further explore the capability of MIL methods, we focused on the higher-level activity captured by individual genes within the tissue, which can be summarized within hallmark pathways underlying cancer initiation and progression. These included processes such as *angiogenesis*, which plays a crucial role in the formation of blood vessels to support tumor growth, *hypoxia*, triggered when cancer cells experience inadequate levels of oxygen, or the *P53 pathway*, which regulates cell-cycle arrest and apoptosis in response to DNA damage (see Table S1 for a complete list). Across 14 key hallmark pathways we observed markedly consistent levels of performance of different MIL algorithms in predicting the expression of the genes involved in the respective pathways (Figure 5).

The E2F target genes had the highest average AUROC in our experiments (Figure 5). This pathway participates in the cell cycle G1/S transition and DNA replication, and is generally upregulated in tumor cells, leading to abnormal cell proliferation.^46^ Such proliferation differences may be more easily captured in the morphology of the cells as well as nuclear staining within H&E-stained sections. In fact, the top 50 highest performing models were for genes involved in cell cycle checkpoints, mitosis and DNA integrity (Table S4), suggesting that cell division-related processes are most easily captured within cancer H&E slides using this methodology. This is not surprising, given the remarkably high performances (>90%) of DL models when it comes to distinguishing tumor areas from normal cells^47^ considering that proliferation is the key defining hallmark of cancer. In contrast, the bottom 50 least performing models (with AUROCs <62%) were for genes involved in tyrosine kinase and apoptotic signaling, nucleotide excision repair and oxidative stress responses (Table S5), which might not be accompanied by visible phenotypic changes in the tumor microenvironment.

Thus, we demonstrate how HistoMIL can be used to assess the detection of thousands of disease-relevant molecules in a speedy and efficient manner, with most informative results obtained for genes that are either expressed throughout the entire slide or not expressed at all within the tissue.

## Discussion

Building a MIL pipeline for WSI datasets is a complex undertaking, demanding extensive engineering expertise and a deep understanding of model hyperparameters. The intricacies of WSIs necessitate additional efforts is terms of data retrieval, preprocessing and normalization compared to typical image processing models used in standard classification tasks. In particular, WSI data requires specialized handling during retrieval and preprocessing due to its unique nature. Moreover, since H&E slides are relatively sensitive to color variations, they require an additional normalization step and should carefully select data augmentation steps (i.e., must not undergo extreme cropping operations). In this paper, we introduce HistoMIL, a Python library designed to streamline the pre- and post-processing of WSIs within a MIL-based pipeline. It seamlessly integrates with PyTorch, a widely used DL toolkit, simplifying the training of a batch of MIL-models for diverse targets. The use of PyTorch Lightning further simplifies the training process. Furthermore, our HistoMIL package offers an uncomplicated method for training a feature extractor using an SSL protocol, which can enhance the performance of various methods within the target data domain. We provide a detailed tutorial on how to use this package, tailored to both technical and non-technical users, in our *Notebooks* folder at https://github.com/secrierlab/HistoMIL.

HistoMIL aims to facilitate the training and application of MIL algorithms for predicting molecular labels based on digital pathology slides by providing an easy-to-use API. In doing so, we hope to equip users with a comprehensive toolkit that empowers them to concentrate on developing new algorithms and addressing biological questions. To achieve this goal, we have implemented a wide range of modules and functions to streamline the process of training and using SSL and MIL. With HistoMIL’s SSL component, generating a feature extractor for the diagnostic slide dataset becomes effortless, subsequently enabling the prediction of various molecular labels. In our experiments, we illustrate how HistoMIL can be used to predict of the expression status of multiple genes in H&E slides using the built-in MIL algorithms. These pipelines have been implemented in the form of interactive notebooks and can be opened and evaluated on cloud platforms such as Google Colab and Kaggle. This highlights how HistoMIL can be used to greatly simplify the complexity of engineering implementations. We hope that the examples provided will assist other users in incorporating MIL methods as effective tools in their analysis pipelines.

By designing HistoMIL to maintain consistency and ease of use when introducing customized models, we have observed that the data processing steps of different algorithms can be shared due to the repeated use of HistoMIL modules. Additionally, different algorithms can also share the same intermediate data results. Furthermore, batch processing and patch aggregation in HistoMIL come with pre-set values, which greatly reduces the level of difficulty for users. It is important to note that HistoMIL is not limited to the implemented MIL algorithms. Due to its modular and scalable design, users can conveniently implement and modify new algorithms. This flexibility allows for the training of customizable algorithms based on existing work. Therefore, any algorithm implementation involving MIL and WSIs can benefit from the functionality we provide. In addition, many tasks involving WSIs can be easily accommodated through modifications to algorithms or loss functions.

We showcased the strong performance of HistoMIL across more than 2,000 gene expression prediction tasks using a variety of MIL algorithms. Notably, we achieved AUCs exceeding 80% for 130 genes. TransMIL demonstrated superior results compared to other MIL or TL algorithms. We showed that amongst classical cancer hallmark pathways the most identifiable within H&E-stained slides are the ones related to cell proliferation, in line with other findings in the field,^10^ whereas kinase signaling, apoptosis and oxidative stress processes are more difficult to capture from the tissue morphology. This suggests that cancer diagnosis and progression tasks could be more easily automated on histopathology slides than tasks related to targeted treatment decisions. Furthermore, the spatial visualization capabilities of HistoMIL pave the way toward further analyses of spatial patterns of gene activity, which could be used to understand how cancers develop within the tissue and interact with their environment.

HistoMIL is an open-source project that will continue to add additional pre-trained models and functionality. In the future, we intend to enhance the currently available models by training them on additional datasets and including other MIL algorithms. A logical extension of our work is its application in tasks like cancer grading, survival prediction and the exploration of other molecular or clinical labels in different tumor types. Importantly, the package is not restricted to the analysis of cancer datasets alone, and could be readily applied to address a wide range of biomedical questions seeking to uncover meaningful patterns within H&E stained slides. Future versions of HistoMIL could aid with patient diagnosis or help pathologists identify subtle differences in gene activity that might not be readily discernible in traditional histological sections. Extracting features and analyzing results is typically a time-consuming and computationally intensive task, but by using HistoMIL we can train models on a very large scale. This may lead to faster and more efficient analysis of whole slide images in future clinical usage.

### Limitations of the study

Our experiments have shed light on certain limitations within the existing MIL algorithms. First, as expected, the performance of these algorithms is highly dependent on the image quality of the slides. As the expression levels of some genes are correlated with the color patterns of the images, the models are sensitive to the color changes in the original slides. In our experiment, to maintain simplicity in the training process and reduce extraneous variables, we did not introduce additional data augmentation steps. This might have compromised the generalization capabilities of the resulting model. In practice, different molecular labels may require distinct data augmentation strategies as critical parameters for performance optimization. We underscore the importance of considering this as an essential step for accurate model construction and prediction.

MIL algorithms also incorporate attention mechanisms and neighborhood information, rendering them more susceptible to variations within a single slide, such as local color changes. Moreover, patch-level predictions can introduce biases. Some genes might not be expressed in all regions of the slide, but because a single expression label is available per slide, the models would tend to assume high expression in all areas when this label is high. We frequently observed this phenomenon in our experiments. Whether this reflects a genuine feature of gene activity within the tissue or a model limitation remains to be determined. To address this issue, future work should focus on incorporating additional annotations, such as those obtained through immunohistochemistry, to acquire higher-resolution data for a specific biomarker across the entire tissue rather than relying on a single label per slide.

The interpretability of an AI model in biology and medical research cannot be overstated. In HistoMIL, the ABMIL and TransMIL implementations inherently feature attention scores to demonstrate good interpretability. The DSMIL method generates patch-level prediction values as part of its output. Furthermore, gradient-based interpretability methods can be incorporated with some simple additional functions. In subsequent extensions, we plan to introduce distinct interpretability components to the HistoMIL framework, thereby providing a unified interpretability interface for all the supported models. To further enhance interpretability, future research endeavors in the field should also incorporate experimental validation of the model predictions.

The number of samples in the tested dataset also limits our analyses. In the context of TL, the number of training samples is determined by the number of WSIs in the dataset multiplied by the number of patches within each WSI. In essence, each patch extracted from a WSI is treated as an independent sample, resulting in a substantial number of training samples. However, when employing the MIL method, the number of training samples for the slide-level classifier is equivalent to the number of WSIs in the dataset. This is because MIL treats each WSI as a single sample, rather than considering each patch within the WSI as an individual sample. This fundamental distinction has the potential to impact the generalization capability of the MIL method. It also makes the model aggregation part prone to being misled by certain samples and thus trapped in local optima. Although we selected the largest cancer dataset available from TCGA (TCGA-BRCA), the total number of WSI samples remains relatively limited, which further constrains the potential performance of our models. Furthermore, while TCGA-BRCA is a cancer dataset, due to time and resource constraints, our current experiment did not include testing on other cancer datasets or those related to other diseases. Thus, our biological conclusions are unlikely to be generalizable to other types of cancers or diseases. Our future work will aim to address those issues by expanding the pool of diagnostic slides from other sources.

## STAR★Methods

### Key resources table

**Table:** 

REAGENT or RESOURCE	SOURCE	IDENTIFIER
**Software and algorithms**		

HistoMIL	This paper	GitHub: https://github.com/secrierlab/HistoMIL; Zenodo: https://doi.org/10.5281/zenodo.8220572
PyTorch	Paszke et al.^34^	https://pytorch.org/
PyTorch Lightning	Falcon^35^	https://lightning.ai/docs/pytorch/stable/
OpenSlide-pytorch	Goode et al.^18^	https://openslide.org/api/python/
ABMIL	Leiby et al.^31^	https://github.com/AMLab-Amsterdam/AttentionDeepMIL
DSMIL	Li et al.^32^	https://github.com/binli123/dsmil-wsi
TransMIL	Shao et al.^36^	https://github.com/szc19990412/TransMIL
TCGAbiolinks	Colaprico et al.^48^	https://bioconductor.org/packages/release/bioc/html/TCGAbiolinks.html
msigdbr	Igor Dolgalev	https://igordot.github.io/msigdbr/
GeneMania	Warde-Farley et al.^49^	https://genemania.org/

**Other**		

H&E slides and RNA-seq data from the TCGA BRCA cohort (GDC data portal)	TCGA, Weinstein et al.^50^	https://www.cancer.gov/tcga; https://portal.gdc.cancer.gov/projects/TCGA-BRCA
MSigDB hallmark gene set	Liberzon et al.^51^	https://www.gsea-msigdb.org/gsea/msigdb/human/genesets.jsp?collection=H

### Resource availability

#### Lead contact

Further information and requests for resources should be directed to and will be fulfilled by the lead contact, Maria Secrier (m.secrier@ucl.ac.uk).

#### Materials availability

This study did not generate new unique reagents.

### Experimental model and study participant details

This paper analyses publicly available RNA-sequencing and H&E data from TCGA, collected from 1,062 breast cancer patients. All data comply with ethical regulations, with approval and informed consent for collection and sharing already obtained by The Cancer Genome Atlas (TCGA). Sex, age and ethnicity information for the study participants can be found at https://portal.gdc.cancer.gov/projects/TCGA-BRCA. Clinical information was available for 1,048 patients, out of which 99% (n = 1,036) were female and 1% (n = 12) were male, with ages comprised between 26 and 90 (median of 58). The cohort was split by ethnicity as follows: 38 Hispanic or Latino, 842 not Hispanic/Latino, 168 unreported. The tumor stages analyzed in the study were as follows: T1 (n = 266), T2 (n = 607), T3 (n = 134), T4 (n = 39), Tx (n = 2). The sex and ethnicity information were not taken into account in the analysis because breast cancer predominantly affects women (99% in the analyzed cohort) and TCGA data is not sufficiently diverse and powered for ethnicity analyses in the context of our study. This may limit the study’s generalisability. Experimental groups were defined by the median expression of each hallmark gene considered in the study as detailed below.

#### Ethics approval and consent to participate

All data employed in this study comply with ethical regulations, with approval and informed consent for collection and sharing already obtained by The Cancer Genome Atlas (TCGA).

### Method details

#### Experimental setting and dataset

In this paper, we exemplify the power and versatility of HistoMIL in oncology-related tasks by building prediction models for 2,487 cancer-related genes.

##### Dataset

The Cancer Genome Atlas (TCGA) is a collaborative project that aims to characterise the genomic and molecular landscape of various cancers.^50^ We chose TCGA-BRCA as the largest available dataset with diagnostic H&E-stained slides and matched RNA-sequencing which we could use to define cancer-relevant labels (n = 2,487). The TCGA-BRCA dataset contains a large amount of data, including whole slide images, genomic data, clinical data, and more. It includes samples from a diverse patient population, including patients of different ages, races, and ethnicities. This helps avoid biases as potential factors that may affect model performance. As a widely used data source, the TCGA-BRCA dataset has undergone quality control, which ensures that the data is of high quality and well-annotated. This can help reduce the noise and variability in the dataset. We downloaded 1,133 diagnostic WSIs from the Genomic Data Commons (GDC) Data Portal.^52^ In our experiment, we split WSIs into patches, then automatically selected WSIs with more than 1,000 patches, and further removed the images which were blurry or containing marks. This left us with 1,012 WSIs for analyses, which we split into 80% for training and 20% for testing.

##### Prediction task

The target labels for the experiments were extracted from the RNA-seq profiles of the same TCGA-BRCA patients for which H&E-stained diagnostic slides were also available, downloaded using the TCGAbiolinks package.^48^ We surveyed 2,487 genes involved in various cancer hallmark pathways derived from MSigDB^51^ using the msigdbr R package (see Table S1), and used the FPKM normalised expression values for each gene to categorise tumors as “highly” or “lowly” expressing the respective gene based on the median split. This is a targeted task designed to demonstrate that HistoMIL can assist researchers in rapidly scaling up the classifiers required for their studies. In this task, the HistoMIL package was used to train and validate prediction models for the expression of the selected 2,487 cancer hallmark genes. In the steps involving MIL training and inference, we employed servers powered by A100 and V100 GPUs. Typically, training for a gene expression spans 100 epochs with early stopping. Predictions for some genes might peak as early as the 5th or 6th epoch, triggering the early stopping mechanism. Within the trainer of HistoMIL, we incorporated options for researchers to either randomly split the training set proportionally or choose K-fold validation, facilitating the validation of the training process. By integrating support for TorchMetrics, users can easily select how to evaluate model performance and monitor training progress. Researchers can also determine which slides, generated by specific hospitals, would be placed in a different set as an independent test set to evaluate model performance. This approach facilitates a more robust evaluation of the model’s generalization capabilities. For this experiment, we used an 80% training set and 20% test set strategy to keep a simple experimental setting. We note that we did not employ the SSL module in this analysis example in order to simplify the training process and to focus on comparing the performance of TL and MIL methods.

Finally, functional enrichment analysis was performed using GeneMania.^49^ By predicting the expression of a gene of interest throughout the entire tissue slide, researchers can highlight the features derived from the attention score or gradient vectors.

We believe using HistoMIL could aid in diagnosis or help pathologists identify subtle differences in gene activity that might not be apparent in traditional histological sections. Extracting features and analysing results is normally a time-consuming and computationally intensive task. By using HistoMIL, we can train models on a very large scale. This may lead to faster and more efficient analysis of whole slide images in future clinical usage.

#### Module design in HistoMIL

##### Data level design

The HistoMIL design includes a data level to handle different data formats, preprocessing and other meta-information of the target cohort. Pre-processing WSIs can be time-consuming and computationally expensive in many cases. By integrating a number of configurations and functions, the HistoMIL package offers a functional interface to smoothly run each necessary step in a different pipeline, and save the intermediate output as needed. After initialising a *Slide* instance and reading the slide files, we divide the preprocessing steps into three separate concept categories: *Tissue*, *Patch*, and *Feature*. Each of these concepts is linked with a parameter class to help users modify the preprocessing steps by following their own experimental requirements. A manager class named *WSI_collector* helps users unify the initialisation, calculation and loading process of these concepts to further decrease the complexity of usage.

###### Reading raw WSI data

HistoMIL can handle different data formats by implementing slide_backend wrappers for widely considered packages such as OpenSlide-Python.^18^ This design can help researchers access the raw data of different WSI formats and provides a common interface for the subsequent steps. For instance, the scale pyramid in WSI processing can be more important than other areas as some WSI files naturally include multi-scale representation of the raw data.^25^ A series of downscaled or upscaled versions of the original slide will help the potential deep learning models get more information. HistoMIL will create a common scale pyramid as metadata of a WSI file. Also, researchers can implement customised slide wrappers to access additional slide formats. This modular design enables the rapid integration of customised slide backends into existing pipelines, thereby facilitating the support of different datasets. All these elements are consolidated within the *Slide* instance when using HistoMIL.

###### Tissue masking and patch extraction

WSIs typically involve a considerable area that only includes non-biologically relevant background elements such as glass or marker pen. HistoMIL includes a *Tissue* class to identify and remove these areas by generating a tissue mask. Inspired by other ML-oriented packages, the *Tissue* class includes a wrapper for an Otsu function to categorise pixels into foreground or background.^28^ Some basic morphological operations are integrated to eliminate small holes within the tissue region. Researchers can also implement different wrapper functions for different purposes. Patch extraction, which aims to decrease the cost of GPU memory when training a model, works in a similar manner. A large WSI can be partitioned into small patches by using an integrated function to iteratively walk through the tissue area. All the intermediate data is stored for further usage, and the proposed implementation can avoid filling available memory with enhanced memory efficiency. By using this scale pyramid, we configure the segmentation process in higher scale pyramid levels as our default setting to decrease computational costs. Similarly, the default settings of patch selection functions, which can decide whether current patches need to be processed in a pipeline, are applied at a high pyramid level. We use a multiple processing pool to further accelerate the patching step, similarly to the CLAM package.^28^ All these parameters (such as step size and window size in the patch concept class) can be easily modified in related parameter classes to fit different requests.

###### Feature extraction

A pre-calculation step for the feature extraction part may decrease time costs during training. Some MIL algorithms^14^,^32^^,^^36^ can work with pre-trained feature extraction networks and choose to calculate feature vectors for each selected patch in a preprocessing step. By following this paradigm, we designed a *Feature* class to synchronise the manipulation and maintenance of the feature vectors generated from each slide. This design can also simplify the potential clustering process for feature vectors within each bag or clustering on the entire dataset. Hence, preprocessing, which would have required the implementation of multiple functions and methods, has been simplified to a few lines of code invocation in HistoMIL (Figure 2A).

##### Model training

In HistoMIL, model training is a crucial part especially for the target molecular labels that may be difficult to identify by only considering tissue morphology differences. MIL has been introduced as a higher performing weakly supervised learning protocol for WSI classification. A slide (or WSI) is considered as a bag of instances in the MIL protocol. Each bag may contain hundreds or thousands of instances (patches) that are assembled into slide-level features for classification. To the best of our knowledge, no existing package to date has been designed to handle MIL methods. There is considerable complexity in building a MIL pipeline for WSI classification tasks due to the inherent complexity of WSI formats, heterogeneous nature of cell morphology, and unbalanced target labels. HistoMIL is designed to simplify the implementation of MIL-based pipelines by implementing Self-Supervised Learning and Multiple Instance Learning modules (Figures 2B and 2C).

###### Self-supervised learning for feature extraction

End-to-End training for a MIL pipeline that consists of feature extractors and aggregators may be computationally expensive, especially in a large WSI. Therefore, the existing model either uses fixed patch features derived from CNNs or simply employs a small number of high-scoring patches to update feature extractors. Inspired by the TL paradigm, the feature extractor network can re-use pre-trained parameters from general image classification domains (e.g., ImageNet). The advantage is that there are different pre-trained feature extraction networks that can be chosen for different tasks. Thus, target classification models can be trained quickly with a pre-trained feature extractor network. However, if a pre-trained feature network is not trained on a WSI dataset, it may require extra effort in the preprocessing steps and the model may not converge. The process of aggregating and classifying may also be susceptible to the issue of overfitting and inadequate supervision, resulting in potential bias and other limitations.

Self-Supervised Learning (SSL) is frequently mentioned as a solution for the mentioned problems. While some studies, such as Lu et al.,^28^ have shown that the SSL phase is a crucial element in a pipeline, integrating the WSI dataset into an SSL pipeline remains difficult. HistoMIL offers an easy way to accomplish this part by using a user-friendly SSL module. To train a feature extractor, the HistoMIL package offers an easy way to apply self-supervised training methods. Firstly, we introduce a trainer class to simplify the training process on a WSI dataset. A wrapper class is created for the timm package^33^ which includes most of the popular backbone architectures and potential pre-trained parameters. Some widely employed SSL methods have been implemented, such as the MoCo^53^^,^^54^ and SimCLR^55^ methods. By using these built-in methods, HistoMIL also offers predetermined hyperparameters to further simplify the training process to new users. Also, it is specifically designed for WSI data. Users can simply download raw data from projects such as TCGA and do not need to worry about preprocessing and data augmentation. In addition, utilising the feature extractor network trained by HistoMIL’s SSL module negates the necessity to account for discrepancies in operator implementation that may arise when importing trained parameters from other packages. Moreover, by embedding SSL methods within the HistoMIL package, researchers can effortlessly incorporate the selection of SSL methods and backbone architectures as hyperparameters when optimising their models.

###### MIL methods

To train a slide-level classifier on target labels, HistoMIL offers a variety of algorithms that are ready to use. To the best of our knowledge, no existing package can simplify the training and classification process for MIL algorithms in WSI datasets. Transfer Learning (TL) protocols and Multiple Instance Learning (MIL) protocols have both been widely considered by researchers for classification tasks on WSIs. While packages such as DeepMed^27^ have been designed to apply TL-based models on WSI datasets, there are considerable difficulties in implementing a MIL model for WSI classification tasks. Whole slide images often encompass several gigabytes or even terabytes, and the aggregation function of MIL methods may suffer due to its high-dimensional feature space, with tens of thousands of feature vectors per slide. Moreover, there is the lack of a standard implementation for various MIL algorithms. For molecular labels, training a MIL model may be even more difficult when faced with problems such as heterogeneity of cell morphology and imbalanced data. In some extreme cases, the target classification model may be vulnerable to overfitting, and is unable to explore rich feature representations because of the insufficient supervised signal. All these challenges require users to have considerable experience with implementing and training deep neural networks. This requirement may exclude researchers who need these models but lack the necessary experience. HistoMIL simplifies this aspect by introducing built-in MIL modules with pre-defined hyperparameters.

In HistoMIL, a customizable sampler function in our Dataloader implementation is introduced that only samples some instances from each bag. Different batch sizes can be used if the MIL algorithm needs to sample N instances from each bag. But if all the patches must be read in at once, the batch size should be fixed at 1 and this may lead to an unstable training process. In our default setting, we chose to decrease the initial learning rate and accumulate gradients over the training steps to smooth the optimisation process. We also include a cohort instance during training to handle patient metadata, which means users can also assign pseudo labels for each patch based on the label per slide, and this enables users to train their model using the transfer learning paradigm as well.

#### Trainer and experiment manager

A significant amount of time and effort is spent on the tuning process and hyperparameter selection steps when training on WSI datasets. We designed the *trainer* class and *experiment* class in HistoMIL, which are built on the PyTorch Lightning framework, to cut these costs. Our target is to simplify the tuning process for different target labels. The SSL, TL and MIL algorithms mentioned above are implemented using a related PyTorch Lightning module in HistoMIL. Researchers can initialise a PyTorch Lightning instance with HistoMIL, and this instance can be easily fed into third-party tuners such as the Ray tuner package.^37^

#### Software and package

HistoMIL utilises Python as the primary implementation language and PyTorch as the underlying deep learning platform.^33^ Using these libraries, HistoMIL can easily implement customised algorithms. Furthermore, we introduced PyTorch Lightning as a higher-level framework to simplify the implementation of training code.^35^ All the built-in algorithms in HistoMIL adhere to PyTorch Lightning’s design philosophy, which decomposes the training process into different functions. This facilitates user customization while also ensuring concise implementation. The HistoMIL package is available at the following GitHub repository: https://github.com/secrierlab/HistoMIL. The version of the code used to produce the results of the paper has been deposited at Zenodo (https://doi.org/10.5281/zenodo.8220572).

#### Development environment

In the present research, we employed the PyTorch framework due to its renowned flexibility and accessibility for deep learning tasks. The computational experiments were executed on a cluster equipped with NVIDIA A100 or V100 graphics cards and 1TB of storage. Our system runs on the Ubuntu 18.04 operating system, with the Anaconda platform facilitating Python-based development. Additionally, to enhance the training efficiency of our deep learning models, GPU acceleration was leveraged using the CUDA toolkit provided by NVIDIA.

### Quantification and statistical analysis

#### Evaluation metrics

To facilitate a robust comparison with state-of-the-art predictors, we adopted the Area Under the Receiver Operating Characteristic Curve (AUROC) as our primary performance evaluation criterion to validate the efficacy of our model. The dataset was randomly partitioned into training and test sets in an 8:2 ratio. Classification tasks for all datasets utilized in this study pertained to binary classification based on varying levels of gene expression. The AUROC metric encapsulates both sensitivity and specificity of predictions. Generally, a higher AUC score indicates superior classifier performance for a given task. An AUC score of 0.5 signifies random guessing, whereas a score of 1 denotes perfect classification.

The pathway enrichment analysis was performed via a hypergeometric test as implemented in GeneMania. All enriched pathways with an FDR>0.1 were taken into account.

## Data Availability

• This paper analyzes existing, publicly available data generated by the TCGA Research Network: https://www.cancer.gov/tcga. The link to the datasets is listed in the key resources table. • The HistoMIL package is available at GitHub: https://github.com/secrierlab/HistoMIL. The version of the code used to produce the results of the paper has been deposited at Zenodo: https://doi.org/10.5281/zenodo.8220572. • Any additional information required to reanalyze the data reported in this paper is available from the lead contact upon request.

## Supplemental information

Document S1. Tables S2, S3 and S5

Table S1. Cancer hallmark genes and pathways considered in the analysis, related to STAR Methods The gs categories are retrieved from MSigDB.

Table S4. Functional enrichment analysis for the top 50 genes in the models, related to Figure 3C The results were obtained using GeneMania and considering a neighborhood of maximum 20 connecting genes in addition to the given gene set.
