# Supplementary material for: HistoMIL: A Python package for training multiple instance learning models on histopathology slides
Source: iScience. 2023 Sep 27;26(10):108073. doi: 10.1016/j.isci.2023.108073 (PMC10583115; doi:10.1016/j.isci.2023.108073)
Supplement: Supplementary file 1 — Document S1. Tables S2, S3 and S5 [file mmc1.pdf]

iScience, Volume *26*

Supplemental information

HistoMIL: A Python package for training multiple instance learning models on histopathology slides

Shi Pan and Maria Secrier

Table S2. Top 30 highest ranked genes in the models, related to Figure 3c. The average AUROC for the TransMIL model is shown.

ENSEMBL ID	AUROC	Methods	Rank	Gene name
ENSG00000164109	0.8569864	TransMIL	1	MAD2L1
ENSG00000164220	0.84697109	TransMIL	2	F2RL2
ENSG00000087586	0.84673154	TransMIL	3	AURKA
ENSG00000142945	0.84377038	TransMIL	4	KIF2C
ENSG00000131808	0.84270191	TransMIL	5	FSHB
ENSG00000087245	0.84103656	TransMIL	6	MMP2
ENSG00000133110	0.83633596	TransMIL	7	POSTN
ENSG00000113721	0.83530128	TransMIL	8	PDGFRB
ENSG00000138778	0.83411038	TransMIL	9	CENPE
ENSG00000159388	0.83244026	TransMIL	10	BTG2
ENSG00000106331	0.83139205	TransMIL	11	PAX4
ENSG00000127920	0.83044714	TransMIL	12	GNG11
ENSG00000094804	0.82824671	TransMIL	13	CDC6
ENSG00000091651	0.82676005	TransMIL	14	ORC6
ENSG00000152583	0.82672584	TransMIL	15	SPARCL1
ENSG00000100526	0.82669723	TransMIL	16	CDKN3
ENSG00000182481	0.82657135	TransMIL	17	KPNA2
ENSG00000085563	0.82619667	TransMIL	18	ABCB1
ENSG00000119699	0.82604635	TransMIL	19	TGFB3
ENSG00000049249	0.82488084	TransMIL	20	TNFRSF9
ENSG00000024526	0.8247695	TransMIL	21	DEPDC1
ENSG00000149557	0.82445121	TransMIL	22	FEZ1
ENSG00000271503	0.82425678	TransMIL	23	CCL5
ENSG00000204262	0.82403779	TransMIL	24	COL5A2
ENSG00000131471	0.82232141	TransMIL	25	AOC3
ENSG00000197170	0.82049513	TransMIL	26	PSMD12
ENSG00000123975	0.81989968	TransMIL	27	CKS2
ENSG00000145604	0.81988764	TransMIL	28	SKP2
ENSG00000160801	0.81949854	TransMIL	29	PTH1R
ENSG00000181104	0.81949687	TransMIL	30	F2R

Table S3. Bottom 50 lowest ranked genes in the models, related to Figure 3c. The AUROC for the TransMIL model is shown.

ENSEMBL ID	AUROC	Methods	Rank	Gene name
ENSG00000135070	0.62306666	TransMIL	2163	ISCA1
ENSG00000196975	0.62282872	TransMIL	2164	ANXA4
ENSG00000187122	0.62188685	TransMIL	2165	SLIT1
ENSG00000076604	0.62057209	TransMIL	2166	TRAF4
ENSG00000091073	0.62016785	TransMIL	2167	DTX2
ENSG00000171815	0.61993241	TransMIL	2168	PCDHB1
ENSG00000184047	0.61934102	TransMIL	2169	DIABLO
ENSG00000134899	0.61919248	TransMIL	2170	ERCC5
ENSG00000138821	0.61907768	TransMIL	2171	SLC39A8
ENSG00000083635	0.61836362	TransMIL	2172	NUFIP1
ENSG00000213614	0.61809361	TransMIL	2173	HEXA
ENSG00000094914	0.61755276	TransMIL	2174	AAAS
ENSG00000160948	0.61684793	TransMIL	2175	VPS28
ENSG00000138798	0.61554384	TransMIL	2176	EGF
ENSG00000149547	0.61537838	TransMIL	2177	EI24
ENSG00000150456	0.61519742	TransMIL	2178	EEF1AKMT1
ENSG00000142089	0.61510062	TransMIL	2179	IFITM3
ENSG00000067057	0.61473215	TransMIL	2180	PFKP
ENSG00000086827	0.61423194	TransMIL	2181	ZW10
ENSG00000083642	0.61228883	TransMIL	2182	PDS5B
ENSG00000196664	0.61196339	TransMIL	2183	TLR7
ENSG00000007350	0.61187065	TransMIL	2184	TKTL1
ENSG00000197879	0.60994864	TransMIL	2185	MYO1C
ENSG00000106399	0.60819638	TransMIL	2186	RPA3
ENSG00000123700	0.6066097	TransMIL	2187	KCNJ2
ENSG00000110395	0.60640025	TransMIL	2188	CBL
ENSG00000102974	0.60604703	TransMIL	2189	CTCF
ENSG00000143384	0.60575247	TransMIL	2190	MCL1
ENSG00000110925	0.60432971	TransMIL	2191	CSRNP2
ENSG00000159173	0.60418701	TransMIL	2192	TNNI1
ENSG00000136159	0.60019696	TransMIL	2193	NUDT15
ENSG00000169908	0.59966278	TransMIL	2194	TM4SF1
ENSG00000176022	0.59714103	TransMIL	2195	B3GALT6
ENSG00000104884	0.59634149	TransMIL	2196	ERCC2
ENSG00000185885	0.59615463	TransMIL	2197	IFITM1
ENSG00000153002	0.59460676	TransMIL	2198	CPB1
ENSG00000048162	0.58932847	TransMIL	2199	NOP16
ENSG00000174837	0.58578765	TransMIL	2200	ADGRE1
ENSG00000162734	0.5853883	TransMIL	2201	PEA15

ENSG00000129682	0.58520699	TransMIL	2202	FGF13
ENSG00000132676	0.58102077	TransMIL	2203	DAP3
ENSG00000169715	0.58059794	TransMIL	2204	MT1E
ENSG00000136167	0.57932603	TransMIL	2205	LCP1
ENSG00000080910	0.5765422	TransMIL	2206	CFHR2
ENSG00000171401	0.55916232	TransMIL	2207	KRT13
ENSG00000104825	0.55896556	TransMIL	2208	NFKBIB
ENSG00000115694	0.54093564	TransMIL	2209	STK25
ENSG00000173039	0.53430718	TransMIL	2210	RELA
ENSG00000163209	0.52865088	TransMIL	2211	SPRR3
ENSG00000164708	0.49522996	TransMIL	2212	PGAM2

Table S5. Functional enrichment analysis for the bottom 50 genes in the models, related to Figure 5. The results were obtained using GeneMania and considering a neighbourood of maximum 20 connecting genes in addition to the given gene set.

Function	FDR	Genes in network	Genes in genome
epidermal growth factor receptor signaling pathway	0.003989663	6	80
negative regulation of ERBB signaling pathway	0.003989663	5	45
regulation of ERBB signaling pathway	0.003989663	6	86
extrinsic apoptotic signaling pathway	0.004244424	7	146
regulation of epidermal growth factor-activated receptor activity	0.005796543	4	24
regulation of epidermal growth factor receptor signaling pathway	0.016265448	5	70
nucleotide-excision repair, DNA incision	0.021746735	4	37
ERBB signaling pathway	0.021746735	6	133
negative regulation of epidermal growth factor receptor signaling pathway	0.029947849	3	14
negative regulation of protein tyrosine kinase activity	0.029947849	3	14
cellular response to oxidative stress	0.034590121	6	156
response to UV	0.038243016	5	96
negative regulation of signaling receptor activity	0.051118903	3	18
regulation of extrinsic apoptotic signaling pathway	0.065961474	5	111
regulation of cysteine-type endopeptidase activity	0.088541534	6	195
